# Demonstration of a pseudo-magnetization based simultaneous write and read operation in a Co_60_Fe_20_B_20_/Pb(Mg_1/3_Nb_2/3_)_0.7_Ti_0.3_O_3_ heterostructure

**DOI:** 10.1038/s41598-020-67776-y

**Published:** 2020-07-01

**Authors:** Tingting Shen, Vaibhav Ostwal, Kerem Y. Camsari, Joerg Appenzeller

**Affiliations:** 1 Department of Physics and Astronomy, Purdue University, West Lafayette, IN 47907 USA; 2 School of Electrical and Computer Engineering, Purdue University, West Lafayette, IN 47907 USA; 3 Birck Nanotechnology Center, Purdue University, West Lafayette, IN 47907 USA

**Keywords:** Ferromagnetism, Spintronics

## Abstract

Taking advantage of the magnetoelectric and its inverse effect, this article demonstrates strain-mediated magnetoelectric write and read operations simultaneously in Co_60_Fe_20_B_20_/Pb(Mg_1/3_Nb_2/3_)_0.7_Ti_0.3_O_3_ heterostructures based on a pseudo-magnetization µ ≡ m_x_^2 ^− m_y_^2^. By applying an external DC-voltage across a (011)-cut PMN-PT substrate, the ferroelectric polarization is re-oriented, which results in an anisotropic in-plane strain that transfers to the CoFeB thin film and changes its magnetic anisotropy H_k_. The change in H_k_ in-turn results in a 90° rotation of the magnetic easy axis for sufficiently high voltages. Simultaneously, the inverse effect is employed to read changes of the magnetic properties. The change of magnetization in ferromagnetic (FM) layer induces an elastic stress in the piezoelectric (PE) layer, which generates a PE potential that can be used to readout the magnetic state of the FM layer. The experimental results are in excellent qualitative agreement with an equivalent circuit model that considers how magnetic properties are electrically controlled in such a PE/FM heterostructure and how a back-voltage is generated due to changing magnetic properties in a self-consistent model. We demonstrated that a change of easy axis of magnetization due to an applied voltage can be directly used for information processing, which is essential for future ME based devices.

## Introduction

Recently, so-called Magnetoelectric (ME) effects^1–4^ in piezoelectric (PE)/FerroMagnetic (FM) structures have attracted substantial research interest, since these may open a path towards controlling magnetic properties by means of an electric field—rather than currents—to achieve low energy dissipation in the writing process of magnetic memory cells^5–22^. As to the reading process, the Giant Magneto-Resistance (GMR)^18^ or Tunneling Magneto-Resistance (TMR)^1^ is usually utilized to readout the stored magnetic information. However, the readout energy based on magneto-resistive methods is not negligible compared with the extremely low writing energies. In addition, the integration of GMR or Magnetic Tunnel Junction (MTJ) stacks with piezoelectric substrates remains a challenge. This raises the question whether voltages that are used in case of the write operation could also be used in a reverse configuration to read the magnetic information in a more power efficient way. To demonstrate this idea, in this article, we have measured the reciprocal ME effect simultaneously and achieved a strain-mediated magnetoelectric write and read unit in a Co_60_Fe_20_B_20_/Pb(Mg_1/3_Nb_2/3_)_0.7_Ti_0.3_O_3_ heterostructure based on a pseudo-magnetization µ ≡ m_x_^2^-m_y_^2^ rather than a net magnetization m_x_, m_y_ or m_z_. Single crystal PMN-PT with (011) orientation was employed in this work as the piezoelectric substrate for achieving large ME effects due to its large piezoelectric coefficients with d_31_ ~ −3,100 pC/N along the [100] direction and d_32_ ~ 1,400 pC/N along the [01-1] direction^23–29^.

The write operation in the magnetic subsystem is accomplished by applying an external DC-voltage across a (011)-cut PMN-PT substrate. The re-orientation of the ferroelectric polarization results in an anisotropic in-plane strain^16^, which transfers to the CoFeB thin film and changes its H_k_^1,16^. The change in H_k_ in-turn results in a 90° rotation of the magnetic easy axis for high enough voltages. On the other hand, to characterize the inverse effect, an AC magnetic field is simultaneously applied in the hard axis direction of the CoFeB film to induce an AC magnetization change. As a feedback, this AC magnetization change in CoFeB introduces strain in the substrate and generates a readout voltage across the PMN-PT^3,4^. Since the AC magnetization change is dependent on the H_k_ of the CoFeB which is being controlled by the DC voltage applied to the PMN-PT, the AC voltage generated (READ) across the PMN-PT is controlled by the external DC voltage applied (WRITE). Taking advantage of the coupling between the magnetic and electric effects, we have achieved both, electrical write and read operation in CoFeB/PMN-PT heterostructures by modulating the easy axis of the CoFeB film with a DC voltage and reading out this modulation through the detected AC voltage across the PMN-PT substrate. Compared with the work reported in Ref. 3, our measurement technique of the electrical read-out is completely different and is based on an AC excitation of the ferromagnetic film. Unlike Ref. 3, the electrical response from the substrate follows the AC drive of the magnetization at different DC-bias points, which indicates that in our experiment WRITE and READ operations are simultaneously demonstrated, in other words, the electrical voltage that is produced at the output (READ) is instantaneously following the magnetic field drive (WRITE) in our experiment. This is in stark contrast with Ref. 3 that shows WRITE and READ operations sequentially for memory applications.

The need for simultaneous READ and WRITE can be understood by considering potential devices that are not necessarily memory devices. For example, in a field effect transistor the change of channel resistance controlled by an applied gate voltage (WRITE) can be read-out by the source-drain current almost instantaneously. Similarly, it is possible to imagine magnetoelectric devices that generate an output voltage (READ) as a function of magnetization that is being driven by an external input, like a transistor. We discuss the possibility of such a device that can function as a tunable random number generator in Fig. 2c.

Our experimental results that are discussed below are in excellent qualitative agreement with a recently proposed equivalent circuit^4^ that considers how the easy axis of an FM is changed due to an applied voltage and how a back-voltage (${\mathrm{v}}_{\mathrm{m}}$) is generated in the PE due to this change in a self-consistent model. The key novelty of our work is to argue that a change of easy axis of magnetization due to an applied voltage can be directly used for information processing and suggests a different mode of operation from normal magnetic random-access memory (MRAM) technology. This difference is illustrated in Table 1, which shows schematically the bit states 0 and 1.

**Table 1:** Schematic diagram of bit states 0 and 1 in spin-torque devices and magnetoelectric(ME) devices represented by the red and yellow arrows respectively.

	Bit 1	Bit 0
Spin-torque devices	<m_x_> = 1	<m_x_> = − 1
Strain control devices	μ = <m_x_^2^ − m_y_^2^> = 1	μ = <m_x_^2^ − m_y_^2^> = − 1

For nanomagnets with an easy axis in the x-direction, conventional current controlled spin-torque devices store the bit information in the form of a net magnetization i.e. <m_x_> or <− m_x_>^23–25^. However, in voltage-controlled ME devices, although the energy dissipation is much lower than conventional current controlled spin-torque devices, a deterministic net magnetization flip is hard to achieve which hinders its practical applications. In this context, the change of easy axis of the nanomagnet under an applied voltage, which is defined as a pseudo-magnetization µ ≡ m_x_^2^ − m_y_^2^ is proposed to be used in information processing^4^. In this paper, we have demonstrated that voltage-controlled pseudo-magnetization states can be written and read, which is an important step towards future spintronics devices.

## Results and discussion

**Figure 1:**
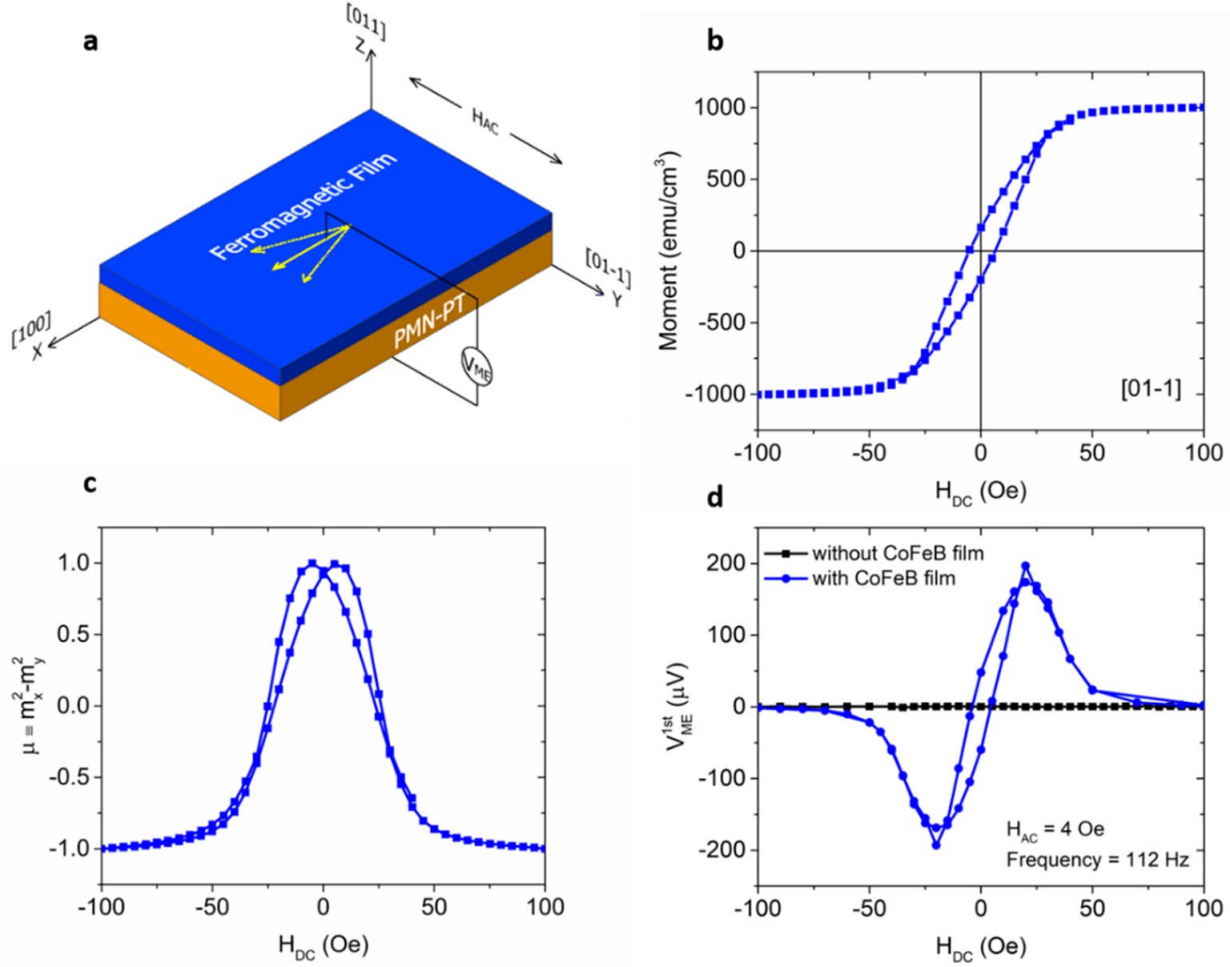
(**a**) Experimental set-up of detecting the pseudo-magnetization electrically. (**b**) Magnetic hysteresis loop of an Au(100 nm)/Ti(10 nm)/(011)-cut PMN-PT(300 µm)/CoFeB(40 nm)/Ta(5 nm) heterostructure measured along the [01-1] direction; (**c**) The corresponding pseudo-magnetization calculated by µ ≡ m_x_^2^ − m_y_^2^. (**d**) The first harmonic magnetoelectric voltage measured across (011)-cut PMN-PT substrates with and without CoFeB film with an AC magnetic field applied in the [01-1] direction. The magnitude and frequency of the AC magnetic field is H_AC_ = 4Oe and f = 112 Hz respectively. The device structure for the black and blue curve is Au(100 nm)/Ti(10 nm)/(011)-cut PMN-PT(300 µm)/Ti(10 nm)/Au(100 nm) and Au(100 nm)/Ti(10 nm)/(011)-cut PMN-PT(300 µm)/CoFeB(40 nm)/Ta(5 nm) respectively.

In this article, electrical detection of the pseudo-magnetization has been achieved in a Co_60_Fe_20_B_20_/Pb(Mg_1/3_Nb_2/3_)_0.7_Ti_0.3_O_3_ heterostructure. Figure 1a shows the schematic configuration of the experimental set-up. The “sloppy” magnetic hysteresis loop measured along the [01-1] direction shown in Fig. 1b indicates the [01-1] direction is the hard axis of the CoFeB film, which is determined by a tensile strain in the [100] direction and a compressive strain in the [01-1] direction induced by the pre-poled (011)-cut PMN-PT substrate. Note that H_DC_ is pointing in the y-direction. The piezo-response of PMN-PT under an external DC voltage and the resulting modulation on the CoFeB magnetization properties are described in Figures S1 and S2 respectively. Figure 1c shows the pseudo-magnetization µ ≡ m_x_^2^ − m_y_^2^ = 1 − 2m_y_^2^ as calculated from the MH loop in Fig. 1b.

**Figure 2:**
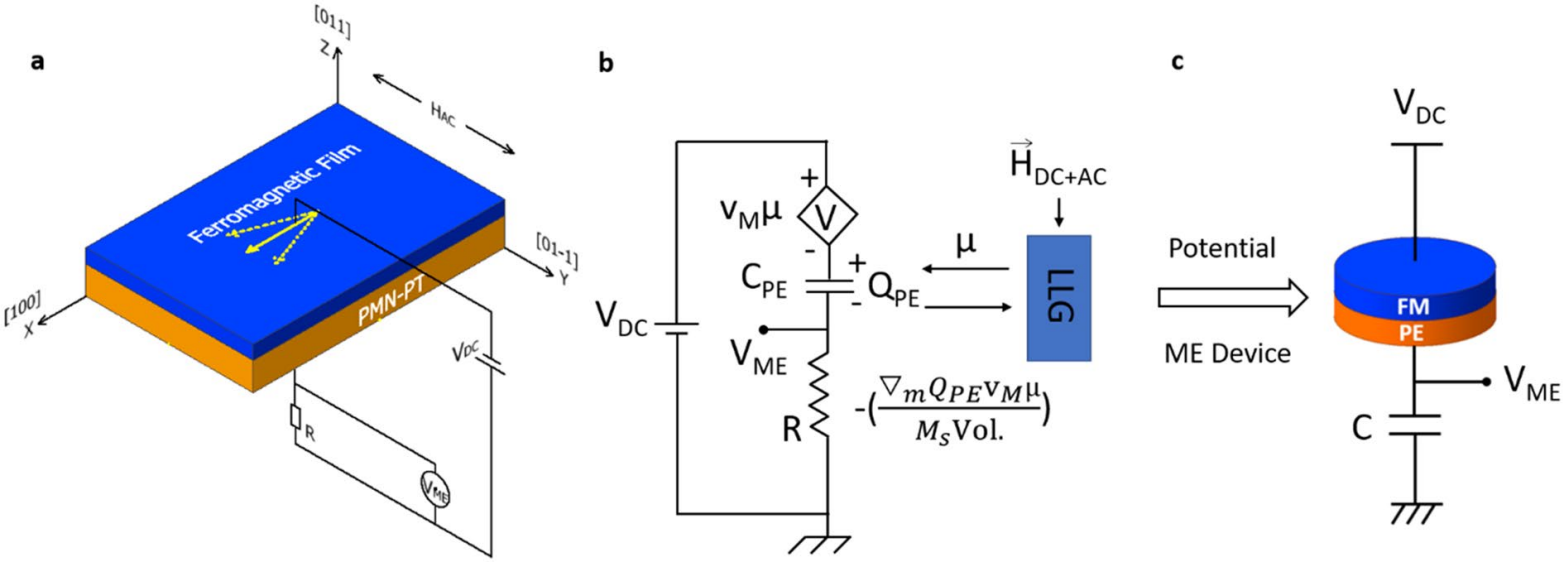
(**a**) Experimental set-up and (**b**) an equivalent circuit model for the PE/FM heterostructure that captures the simultaneous ME write and read operation based on the pseudo-magnetization proposed in Ref. 4. (**c**) A concrete ME device that could be used for practical applications, where the FM is fabricated as a low-barrier nanomagnet (see Ref. 4 for the detailed operation of the voltage-controlled ME device).

To characterize the pseudo-magnetization information by electrical means in the PE/FM heterostructure, an AC magnetic field H_AC_ with an amplitude of 4Oe at a frequency of 112 Hz was applied in addition to a DC magnetic field in the [01-1] direction, which induces an AC magnetization change in the way shown by the yellow arrows in Fig. 1a. The change of magnetization induces deformation in the FM film due to the magnetostriction effect^30^ which transmits to the PE substrate and results in a change of P due to the piezoelectric effect^31^. The variation of P during the reorientation of the magnetization gives rise to a magnetoelectric voltage defined as V_ME_ across the heterostructure. The blue curve in Fig. 1d shows the first harmonic of the magnetoelectric voltage, i.e. $V_{ME}^{1st}$ measured by a lock-in amplifier SR830. For an AC magnetic field of frequency 2π/w, the 1st and 2nd harmonic of V_ME_ is extracted using

1 $${V}_{ME}^{1st}=\frac{1}{T}{\int }_{0}^{T}{V}_{ME}\left(t\right)*\mathrm{s}\mathrm{i}\mathrm{n}\left(wt\right)dt={H}_{AC}*\frac{\partial {V}_{ME}}{\partial {H}_{DC}}$$

2 $${V}_{ME}^{2nd}=\frac{1}{T}{\int }_{0}^{T}{V}_{ME}\left(t\right)*\mathrm{s}\mathrm{i}\mathrm{n}\left(2wt\right)dt={({H}_{AC})}^{2}*\frac{\partial }{\partial {H}_{DC}}\left(\frac{\partial {V}_{ME}}{\partial {H}_{DC}}\right)$$

where T = w/2π is the period of the AC magnetic field. According to the analysis in Ref. 3,4, ${V}_{ME}={v}_{m}\mu$, where v_m_ is the back-voltage constant which will be discussed more later in the context of Fig. 2. Thus,

3 $${V}_{ME}^{1st}={H}_{AC}*\frac{\partial {V}_{ME}}{\partial {H}_{DC}}={H}_{AC}*{v}_{m}*\frac{\partial \mu }{\partial {H}_{DC}}$$

4 $${V}_{ME}^{2nd}={({H}_{AC})}^{2}*\frac{\partial }{\partial {H}_{DC}}\left(\frac{\partial {V}_{ME}}{\partial {H}_{DC}}\right)={\left({H}_{AC}\right)}^{2}*{v}_{m}*\frac{\partial }{\partial {H}_{DC}}\left(\frac{\partial \mu }{\partial {H}_{DC}}\right)$$

Thus, the complicated shape of the detected $V_{ME}^{1st}$ signal shown in the blue curve in Fig. 1d electrically characterizes the slope of µ(H_DC_) in Fig. 1c due to the AC magnetization change (${V}_{ME}^{2nd}$ will be discussed later in the context of Figure S3).

The black curve in Fig. 1d shows the first harmonic magnetoelectric voltage measured with a control sample, which consists of an identical (011)-cut PMN-PT substrate but with Ti (10 nm)/Au (100 nm) deposited on both top and bottom surfaces without a CoFeB film. In this case, we have observed no read-out signal which confirms that the magnetoelectric voltage measured across PMN-PT is induced by the CoFeB film.

Having established the principle of electrical read out of the pseudo-magnetization µ, we will discuss next the simultaneous ME write and read operation in the same PE/FM sample. (For a detailed discussion on the individual READ and WRITE process please see Supplementary Section II and III.) The experimental set-up is shown in Fig. 2a. Before moving on to the actual measurement results, we will discuss the measurement principle based on an equivalent circuit model illustrated in Fig. 2b^4^. This circuit has been experimentally benchmarked against the results reported in Ref. 3.

The magnetization dynamics of the FM layer are calculated from the Landau–Lifshitz–Gilbert equation self-consistently which takes the stress-induced anisotropy field through the charge on the PE capacitor as an input and produces the pseudo-magnetization μ ≡ m_x_^2 ^− m_y_^2^ as an instantaneous output. The circuit equation is given as:

5 $${V}_{DC}=\frac{\partial {E}_{M}}{\partial {Q}_{PE}}+\frac{{Q}_{PE}}{{C}_{PE}}+R\frac{d{Q}_{PE}}{dt}$$

where E_M_ is the magnetic energy, C_PE_ is the capacitance of the PE material, Q_PE_ is the charge on the PE capacitor and R is a resistor connected in series with the PE/FM heterostructure. The write operation is accomplished by an effective field:

6 $$ {\mathop {H}\limits^{ \to }}{}_{ME} = - \frac{{\nabla_{m} E_{M} }}{{M_{S} Vol.}} $$

where ∇_m_ is the gradient operator with respect to the magnetization directions m_i_. M_S_ and Vol. are the saturation magnetization and the volume of the magnetic film respectively. The read operation is accomplished due to a changing “back-voltage” through the pseudo-magnetization μ, ${V}_{ME\_tot}=\frac{\partial {E}_{M}}{\partial {Q}_{PE}}={v}_{m}\mu$, and ${E}_{M}={Q}_{PE}{v}_{m}\mu$.

To measure the magnetoelectric voltage using a lock-in amplifier, a resistor R with a resistance value comparable to the impedance of the piezoelectric capacitor is connected in series with the heterostructure. ${V}_{ME}^{1st}$ and ${V}_{ME}^{2nd}$ measured across R are used to read the magnetic information of the FM layer since they are proportional to the slope and curvature of µ(H_DC_) due to the AC magnetization change respectively as shown in Eqs. (3) and (4). MATLAB analytical simulations on simultaneous ME write and read operations are discussed in Figure S4.

In Fig. 2c, we illustrate the concept of a magntoelectric device that can be used for practical applications by employing a PE/FM stack in series with a capacitor, where the FM is fabricated as a low-barrier nanomagnet whose energy barrier is comparable to the thermal energy *kT*. If the magnet is fabricated as a circular magnet without any uniaxial anisotropy, the magnetization would fluctuate randomly in the plane due to the presence of thermal noise. Due to the back-voltage that would be induced by the fluctuations of magnetization, this 2-terminal device can operate as a voltage controllable tunable random number generator^32–37^ that operates without any external magnetic fields^4^.

Similar to the operation of this conceptual device, in our experiment the change in the magnetization of the FM is turned into changes in the output voltage, with the difference that instead of random fluctuations the magnetization is being deterministically driven by external magnetic fields. In practical applications involving low barrier magnets, such external DC or AC magnetic fields would not be required since magnetization would naturally be driven by thermal noise.

**Figure 3:**
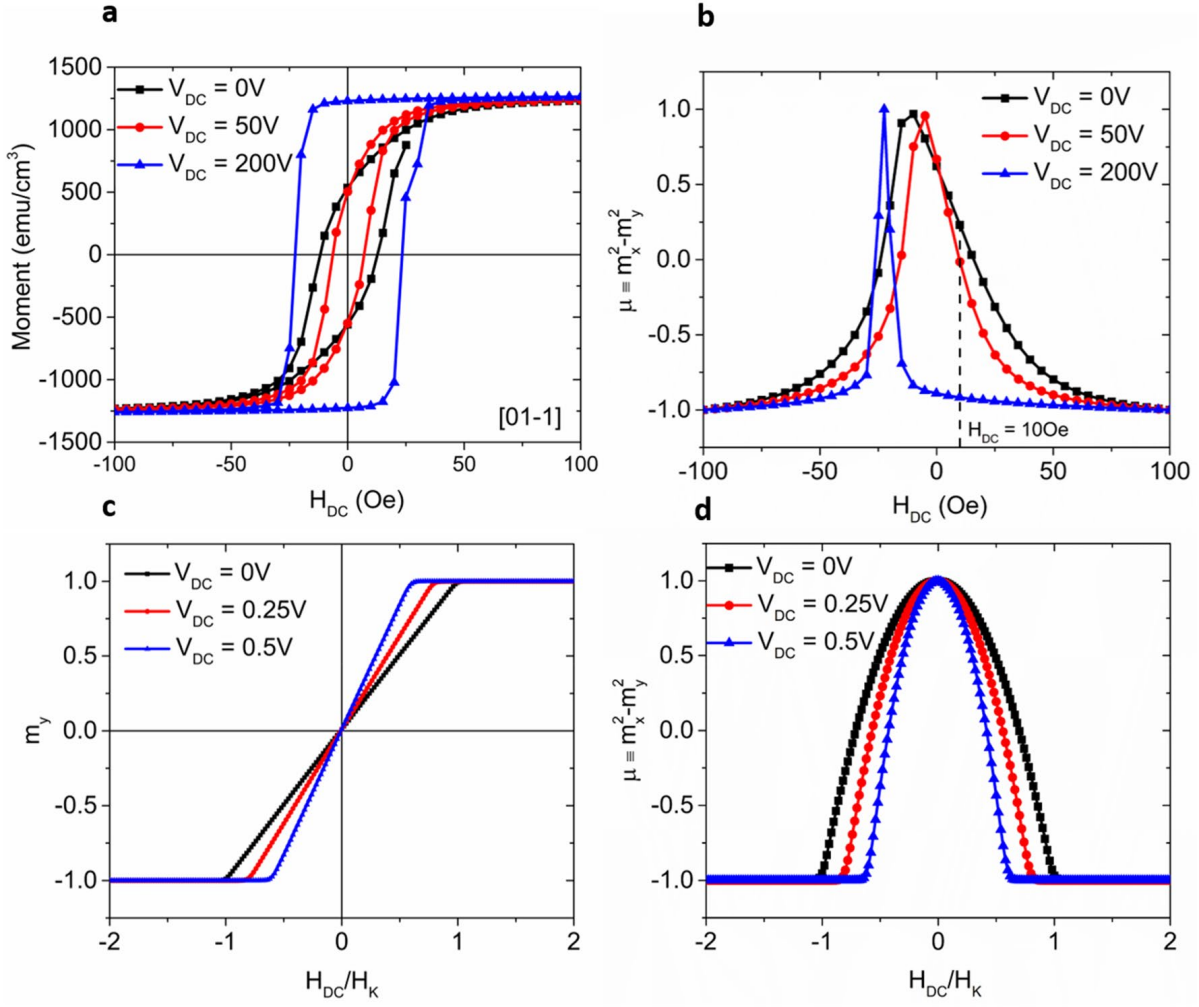
(**a**) M–H loops of an Au(100 nm)/Ti(10 nm)/(011)-cut PMN-PT(300 µm)/CoFeB(40 nm)/Ta(5 nm) heterostructure measured along the [01-1] direction under different voltages. (**b**) The corresponding pseudo-magnetization calculated from the data in (**a**) using µ ≡ m_x_^2^ − m_y_^2^. (c) SPICE simulation results on the average in-plane hard axis magnetization m_y_ as a function of DC magnetic field in the y-direction at different DC voltages (V_DC_). (**d**) The corresponding pseudo-magnetization calculated from µ ≡ m_x_^2^ − m_y_^2^. Without loss of generality, the following parameters are used in the numerical circuit: H_K_ = 25 Oe, Ms = 300 emu/cc, Vol. = 5e−13 cm^3^, damping coefficient α = 0.1, demagnetization field H_D_ is assumed to be 100 H_K_. The piezoelectric capacitance C_PE_ is chosen to be 7.5 pF, and the load resistance is 0.1 Ω.

Experimentally, the PE/FM heterostructure used in this measurement is CoFeB (40 nm)/(011)-cut PMN-PT (300 µm) with Ta and Ti/Au as the top and bottom electrodes respectively. Voltage-controlled magnetization measurements were carried out in a Magnetic Property Measurement System (MPMS) at room temperature. Figure 3a shows the stack’s magnetic hysteresis loops measured along the [01-1] direction under different voltages. In all measurements, “positive DC voltage” means that the bottom electrode has a higher (more positive) potential than the top electrode. With DC voltages applied in the [011] crystalline direction, a strong in-plane anisotropic piezo-strain is induced due to the re-orientation of the ferroelectric polarization (P)^31,38–40^ which transfers to the CoFeB thin film and provides an in-plane magnetic anisotropic field that changes the magnet’s H_k_. When the strain is sufficiently large, the result is a 90° rotation of the magnetic easy axis^1,16^. It is worth mentioning that an interfacial charge-mediated ME coupling effect may also induce magnetization modulations in the FM layer. However, the spatial extent of this interfacial modulation is typically less than 1–2 nm, which is too small to account for the large magnetization modulation observed in our experiment.

As discussed in Fig. S1, under large positive voltages [case 2 in Fig. S1], there is a tensile strain in the [01-1] direction and a compressive strain in the [100] direction, which favors a magnetization along the [01-1] direction and results in a high remnant magnetization as evident from Fig. 3a. Figure 3b shows the calculated pseudo-magnetization when scanning H_DC_ from 100Oe to − 100Oe. It is obvious that both the line width and the slope of µ(H_DC_) have been modulated by V_DC_.

Furthermore, the experimental results are described qualitatively by theoretical simulations using the equivalent circuit shown in Fig. 2b. Figure 3c shows the average magnetization at different input voltages, verifying the easy-axis anisotropy modulation by different input voltages which qualitatively agrees with the experimental results in Fig. 3a. The corresponding pseudo-magnetization µ ≡ m_x_^2^ − m_y_^2^ is shown in Fig. 3d.

Note that we have not attempted a quantitative benchmarking of the experiment, which is why the V_DC_ values in Fig. 3b, d are not the same. Another notable difference between the experiments and simulations is that we assumed the magnetic layer behaves like a monodomain nanomagnet without hysteresis in the hard-axis M-H curves, which results in the same pseudo-magnetization peak positions under different voltages in Fig. 3d. The experiment involves an un-patterned CoFeB film whose magnetization dynamics are very likely dependent on multi-domain behavior. The external voltage modulated hysteresis causes a shift of the pseudo-magnetization peak positions in Fig. 3b. Moreover, in the simulations, we have chosen a much thinner dielectric with a smaller area to keep relatively low capacitances to allow transient, SPICE-level circuit simulations.

Simultaneous with the modulated pseudo-magnetization by the external DC voltage, the magnetoelectric voltage V_ME_ was measured using the experimental set-up shown in Fig. 2a. Experimental results on $V_{ME}^{1st}$ measured across R with an AC magnetic field superimposed on a DC magnetic field along the [01-1] direction when V_DC_ = 0 V, 20 V and 50 V are shown in Figure S5. Figure S5(c) shows simulation results of the normalized in-phase component of the AC voltage $V_{ME}^{1st}$ as a function of DC magnetic fields that is read out from the circuit in Fig. 2b.

**Figure 4:**
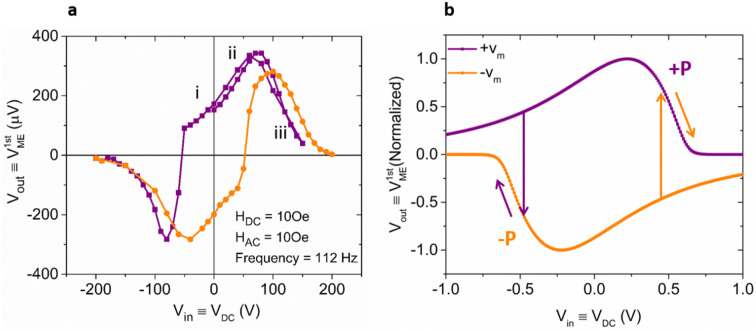
(**a**) Experimental results on the relation between V_in_ ≡ V_DC_ and V_out_ ≡$V_{ME}^{1st}$ when H_DC_ = 10Oe, H_AC_ = 10Oe and the frequency of the AC field is 112 Hz. The purple and orange lines represent scanning V_in_ ≡ V_DC_ from + 200 to − 200 V and from − 200 to + 200 V respectively. (**b**) The purple and orange lines are simulation results on $V_{ME}^{1st}$ as a function of V_DC_ at a fixed DC magnetic field corresponding to 0.5 H_DC_/H_K_ that is read out from the circuit in Fig. 2b when the back-voltage constant is + v_m_ and − v_m_ respectively. Since our simulation does not capture a voltage controlled ferroelectric polarization switching, positive and negative v_m_ values had to be used in the simulation. The arrows indicate how V_out_ versus V_in_ would behave once polarization switching is considered. Note that for the normalized ${V}_{ME}^{1st}$ the actual value of the back-voltage v_m_ does not change the simulation results.

When envisioning a device application, the idea is to use voltage-to-spin conversion for the write operation and spin-to-voltage conversion for the read. Thus, the types of device characteristics that are desirable would have a voltage input V_in_ and voltage output V_out_, with the magnetic information mediating between the two. To show how an input DC voltage, can control indeed in our devices a V_ME_ output voltage, we have fixed H_DC_ at 10Oe and have swept V_in_ between − 200 and 200 V. As shown in Fig. 4a, b, the relationship between V_in_ ≡ V_DC_ and V_out_ ≡$V_{ME}^{1st}$ are non-monotonic in both experiment and SPICE-simulations. This is the case since the slope of µ(H_DC_) which represents the magnitude of $V_{ME}^{1st}$ is different for different V_DC_-values due to the change of H_k_ in the CoFeB film according to the explanation in Fig. 3. For example, in Fig. 3b, when H_DC_ is fixed at 10Oe, the slope of µ increases when V_DC_ increases from 0 to 50 V and then decreases when V_DC_ increases to 200 V. Thus, for the states (1) through (3) in Fig. 4a, one obtains a maximum in $V_{ME}^{1st}$ for (2). The abrupt switch in both the purple and orange lines around + /− 50 V corresponds to the piezoelectric polarization (P) switching when scanning V_DC_. The relation between the piezoelectric polarization P and $V_{ME}^{1st}$ is shown in Fig. S6 indicating $V_{ME}^{1st}$ (P) = − $V_{ME}^{1st}$ (− P).

Figure 4b illustrates simulation results on the normalized $V_{ME}^{1st}$ as a function of V_DC_ at a fixed DC magnetic field corresponding to 0.5 H_DC_/H_K_ that is read out from the circuit in Fig. 2b. Note that to capture the hysteretic nature of $V_{ME}^{1st}$ versus V_DC_ in the simulation, the coefficient v_m_ is made negative when the polarization switched. Compared with Fig. 4a, all the experimental results are in good qualitative agreement with those predicted by the circuit model.

Next, to extract the back-voltage constant in our experimental heterostructure, we performed a quantitative benchmarking of our results. Experimentally, when V_in_ = 50 V, $V_{ME}^{1st}$ = 290 µV with H_AC_ = 10Oe and H_k_ = 70Oe read from the M–H loop shown in Fig. 3a. SPICE simulations were performed after considering the circuit loss factor $\frac{{\left| {jwRC} \right|}}{{\left| {jwRC + 1} \right|}} = 0.37$ in the experimental set-up and all other experimental parameters. The coefficient v_m_ was calculated to be 5.5 mV according to Eq. (1) and the analysis in the context of Fig. 2 to match the experimental value of $V_{ME}^{1st}$ = 290 µV when $\frac{{H_{AC} }}{{H_{k} }} = \frac{10Oe}{{70Oe}}$. v_m_ is a measure of the particular coupling strength between the chosen PE and FM materials. Based on two assumptions that: (1) lossless and elastic strain transfer from the FM to the PE and (2) the thickness of PE layer being much larger than the FM thickness, a theoretical value v_m_ can be calculated from $v_{m} = \frac{{Bdt_{FM} }}{2\varepsilon }$ using the thickness of the FM layer t_FM_ = 40 nm, the magneto-elastic constant B = − 4MPa^41^, the net PE constant d = d_31_ – d_32_ = 4,500 pm/V, and the permittivity of the PE layer ε = 600ε_0_^4^^.^The estimated v_m_ = 70 mV is about one order of magnitude higher than the one extracted from the experimental results. As a point of reference, from the experimental results in Ref. 3 on [N*(TbCo_2_/FeCo)]/PMN-PT (N is the layer number) a v_m_ of 37 mV can be extracted according to the analysis in Ref. 4. Using $v_{m} = \frac{{Bdt_{FM} }}{2\varepsilon }$ on the other hand, one finds v_m_ = 49 mV for the experiment in Ref. 3. While in both cases the experimentally extracted ${\mathrm{v}}_{\mathrm{m}}$ is lower than the calculated one, we believe that the larger discrepancy in our case is likely a result of the non-ideal interface coupling between CoFeB and PMN-PT layers which may be influenced by a variety of factors. For example, the Joule magnetostriction associated with domain movements in the FM layer plays an important role in determine the strength of interface coupling. Since the ME effects involves dynamic magneto-mechanical coupling, unimpeded domain-wall motion, domain rotation are key requirements for the FM layer. Thus, a soft, high initial permeability and high magnetostriction ferromagnetic layer is preferred for strong ME effects. Moreover, the interface quality between the PE and FM layers is also important. This is because defects, inhomogeneities, grain boundaries and growth-induced stress may pin the domain and limit wall motion and rotation. In summary, to achieve a high v_m_ in PE/FM heterostructures, a magnetic layer with favorable domain dynamics and an interface free of growth-induced stress or defects are desired in practical applications^42,43^.

In conclusion, taking advantage of the coupling between magnetic and electric effects, we have achieved both, electrical write and read operation in CoFeB/PMN-PT heterostructures simultaneously based on a voltage tunable pseudo-magnetization where V_in_ = 0 V corresponds to state 1 (the case when the easy axis of the CoFeB film is in the x-direction) and V_in_ =  ± 200 V corresponds to the state 0 (the case when the easy axis of the CoFeB film is in the y-direction). Since the strain induced in the PMN-PT is proportional to the electrical field applied across the structure, a 30 nm film would allow decreasing the write voltage V_in_ to ± 20 mV, making our approach attractive for various magnetic device applications. Moreover, our experimental results are qualitatively consistent with the theoretical simulations of an equivalent circuit reported in Ref. 4. In this work, a novel magnetic operation mode is proposed where a pseudo-magnetization $\mu = m_{x}^{2} - m_{y}^{2}$ rather than a net magnetization m_x_, m_y_ or m_z_ is used as the bit states in MRAM technology. The experimental and theoretical work reported here promises the feasibility of magnetic field free magneto-electric read and write operations in a magnetic system by utilizing a PE/FM stack using a low-barrier nano-magnet to obtain an ultra-low power tunable random number generator^4^, which is significant for the development of voltage-controlled spintronics and low power, high-speed data storage technology.

## Methods

### Fabrication

Amorphous CoFeB films were deposited on one side of the poled (011)-cut PMN-PT substrate in a multisource magnetron sputtering system with a base pressure of 3E-8 Torr. After this, a 5 nm Ta capping layer was deposited in the same chamber. On the other side of the PMN-PT substrate, a 10 nm Ti seeding layer was deposited in a e-beam evaporation system followed by a 100 nm Au film as a bottom electrode of the PE/FM heterostructure.

### SPICE simulation

The equivalent circuit SPICE model calculates a time dependent magnetization and voltage, allowing us to replicate even dynamics of the experimental measurement self-consistently. The following parameters of the ferromagnetic layer were used in the numerical circuit: H_K_ = 25 Oe, Ms = 300 emu/cc, Vol. = 5e−13 cm^3^, damping coefficient α = 0.1, demagnetization field H_D_ is assumed to be 100 H_K_. The piezoelectric capacitance C_PE_ is chosen to be 7.5 pF, and the load resistance is 0.1 Ω. To calculate the ME voltage, first a constant DC field is applied to the FM in the in-plane hard axis [01-1] direction during the entirety of the transient simulation (10 ns for the present case). After the first 5 ns to allow for the magnet dynamics to reach a steady state, an AC magnetic field with a 500 MHz frequency and amplitude of 0.1 H_K_ is applied along the same direction.

## Supplementary information

Supplementary file 1 (DOCX 1870 kb)
