# Supplementary material for: Demonstration of a pseudo-magnetization based simultaneous write and read operation in a Co60Fe20B20/Pb(Mg1/3Nb2/3)0.7Ti0.3O3 heterostructure
Source: Sci Rep. 2020 Jul 1;10:10791. doi: 10.1038/s41598-020-67776-y (PMC7329837; doi:10.1038/s41598-020-67776-y)
Supplement: Supplementary file 1 — (DOCX 1870 kb) [file 41598_2020_67776_MOESM1_ESM.docx]

Demonstration of a pseudo-magnetization based simultaneous write and read operation in a Co_60_Fe_20_B_20_/Pb(Mg_1/3_Nb_2/3_)_0.7_Ti_0.3_O_3_ heterostructure

Tingting Shen *^‡ 1,3^, Vaibhav Ostwal*^‡2,3^, Kerem Y. Camsari^2^, Joerg Appenzeller^2,3^

1. Department of Physics and Astronomy, Purdue University, West Lafayette, Indiana 47907, United States

2. School of Electrical and Computer Engineering, Purdue University, West Lafayette, Indiana 47907, United States

3. Birck Nanotechnology Center, Purdue University, West Lafayette, Indiana 47907, United States

^‡^. Authors contributed equally to the article

I: WORKING PRINCIPLE OF (011)-CUT PMN-PT


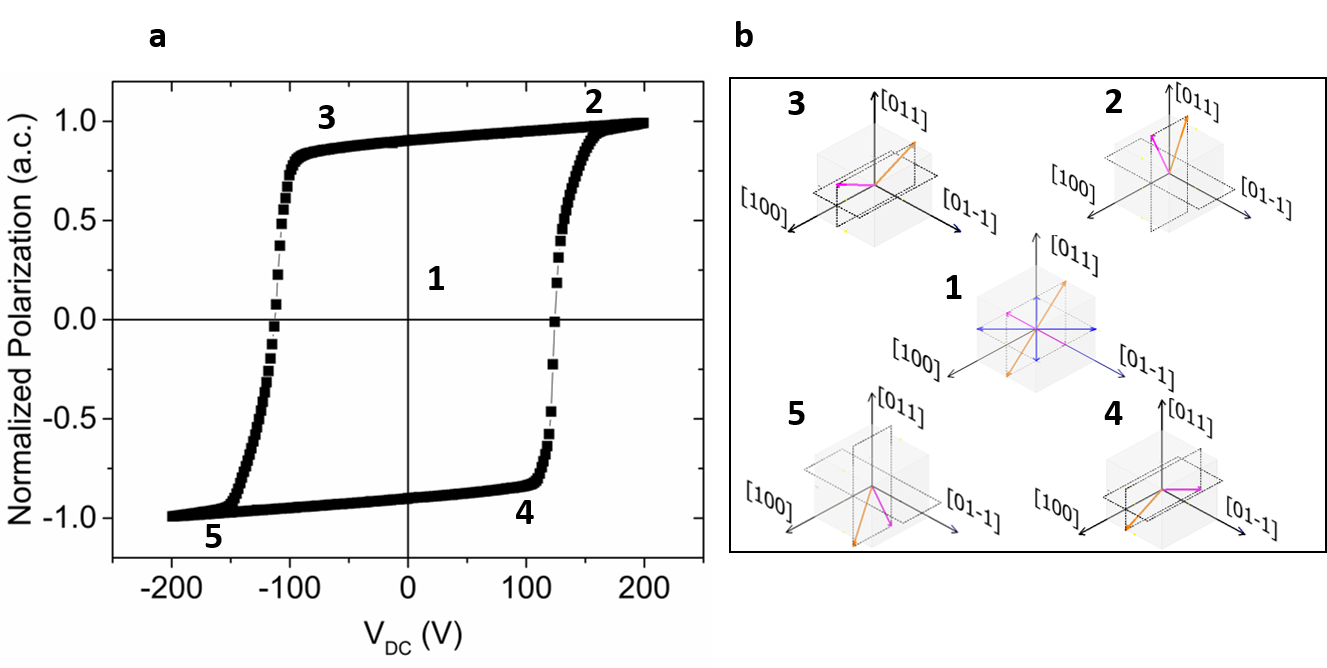


FIG. S1. (a) Experimentally measured ferroelectric polarization versus DC voltage properties of the PMN-PT. (b) The unit cell and spontaneous ferroelectric polarizations of (011)-cut PMN-PT correspond to different cases labeled by 1-5 in (a).

Single crystal PMN-PT with (011) orientation was employed in this work as the piezoelectric substrate for achieving large ME effects due to its large piezoelectric coefficients with d_31_ ~ -3100 pC/N along the [100] direction and d_32_ ~ 1400 pC/N along the [01-1] direction [1-4]. With DC voltages applied in the [011] crystalline direction, a strong in-plane anisotropic piezo-strain is induced due to the re-orientation of the ferroelectric polarization (P) [5-8]. As shown in Fig. S1(a), the PMN-PT exhibits a typical square-shaped ferroelectric hysteresis behavior at room temperature. The inserts one through five in Fig. S1(b) represent the unit cell and spontaneous ferroelectric polarizations of (011)-cut PMN-PT under different DC voltages in accordance with the 1-5 states labeled in Fig. S1(a) respectively.

For PMN-PT with rhombohedral (R) phase, the spontaneous polarizations are along the [111] directions, which means the diagonals of the (011) and (01-1) plane in the (011)-cut crystal. Before poling, the polarization orientations are randomly distributed in the eight possible directions and the total polarization P = 0, as shown in case 1. In this situation, there is no net strain in the PMN-PT. When the PMN-PT is poled along the [011] direction by a large positive voltage, a net polarization in the [011] direction occurs as a result of the only two remaining polarization vectors – yellow and pink in Fig. S1(b). If compared to the two initial polarization vectors with a positive component in the [011] direction as displayed in case 1, case 2 exhibits a larger net polarization of these two vectors, leading to a compressive strain in the [100] direction and a tensile strain in the [01-1] direction. After removing the electric field and applying a small negative voltage that is not large enough to switch the polarization to the other direction, the net polarization is still in the [011] direction but its magnitude is smaller than the sum of the two initial polarization vectors (pink and yellow) with positive component in the [011] direction. Thus, if compared with the un-poled state, the crystal is elongated in the [100] direction and compressed in the [01-1] direction as shown in insert 3. Similarly, when the PMN-PT is poled along the negative [011] direction by a sufficiently large negative voltage, a net polarization that points in the negative [011] direction is obtained as shown in insert 5. This state is characterized, like case 2, by a compressive strain in the [100] direction and a tensile strain in the [01-1] direction. Finally, by removing the negative voltage and applying a small positive voltage, state 4 is reached that is characterized by the same situation as case 3. The anisotropic in-plane strain that can in this way be produced by applying a voltage to the PMN-PT crystal transfers to the CoFeB thin film and provides an in-plane magnetic anisotropic field that changes the magnet’s H_k_.

II: ME WRITE OPERATION


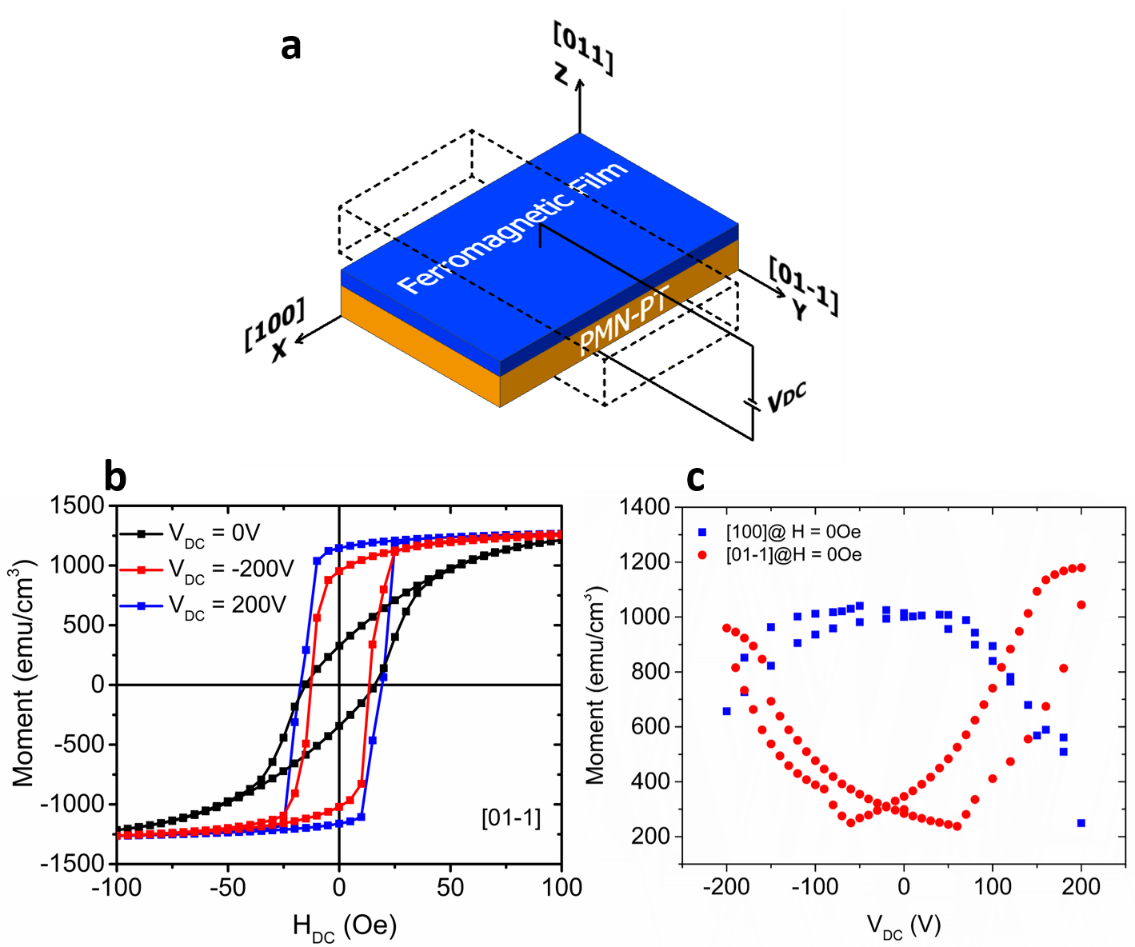


FIG. S2. (a) Schematic configuration of the ferromagnetic/piezoelectric heterostructure and the piezo-response of PMN-PT under a sufficiently positive or negative DC voltage. (b) Magnetic hysteresis loops of an Au(100nm)/Ti(10nm)/(011)-cut PMN-PT(300µm)/ CoFeB(20nm)/Ta(5nm) heterostructure measured along the [01-1] direction under different voltages. (c) Voltage-control of magnetization measured along both [100] and [01-1] directions with H = 0Oe.

The magnetoelectric (ME) write operation has been achieved in a Co_60_Fe_20_B_20_/ Pb(Mg_1/3_Nb_2/3_)_0.7_Ti_0.3_O_3_ heterostructure. Fig. S2(a) shows the schematic configuration and the piezo-response of PMN-PT under a sufficiently positive or negative DC voltage. Magnetic hysteresis loops of a PE/FM heterostructure consisting of a PMN-PT crystal and a 20nm CoFeB layer with in-plane magnetic anisotropy (IMA) measured along the [01-1] direction under different voltages are shown in Fig. S2(b). As discussed in Fig. S1 and Fig. 3(a) in the main text, large positive and negative voltages [case 2 and 5 in Fig. S1] induces a tensile strain in the [01-1] direction and a compressive strain in the [100] direction. Thus, the magnetization along the [01-1] direction becomes easier as shown in Fig. S2(b). When the voltage is removed, the compressive strain in the [01-1] direction and tensile strain in the [100] direction makes magnetization along the [01-1] direction harder. Simultaneously, for the [100] direction, magnetization is easier for zero DC voltage and harder for large positive and negative voltages. Fig. S2(c) shows the change of remnant magnetization according to the applied DC voltages along both [100] and [01-1] directions, clearly indicating the complementary trend, i.e. decreasing moment towards large electric fields in the blue compared to increasing moment towards large electric fields in the red measurement case. Both exhibit a butterfly-like behavior, which is in consistent with the butterfly-like trend of induced in-plane strain in the PMN-PT [9].

III: ME READ OPERATION


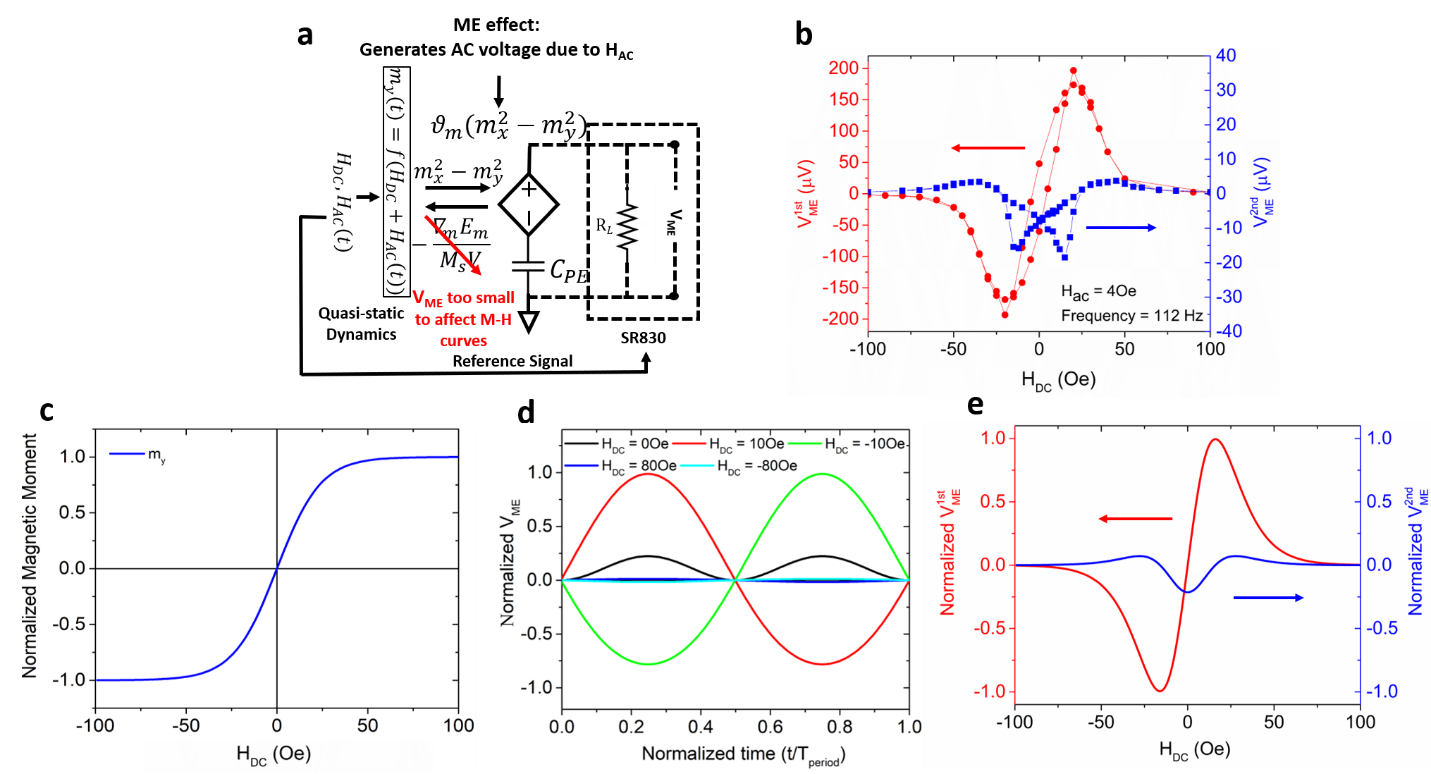


FIG. S3. (a) Circuit model of the ME read operation. (b) Experimental results on $V_{ME}^{1st}$ and $V_{ME}^{2nd}$measured in the experimental set-up shown in Fig. 1(a). Analytical results on (c) the M-H loop in the [01-1] direction without external DC voltages; (d) Normalized total V_ME_ within one period of an AC magnetic field with a magnitude of H_AC_ = 4Oe and frequency of 112Hz in the Y direction at different DC magnetic fields; and (e) the corresponding $V_{ME}^{1st}$ and $V_{ME}^{2nd}$.

As discussed in the main text, the ME effect can be used to read the magnetic state in the CoFeB film due to the magnetostriction effect [1] and the piezoelectric effect [5]. Figure S3(a) shows the corresponding circuit model. The red and blue curves in Fig. S3(b) are the first and second harmonic magnetoelectric voltage, i.e. $V_{ME}^{1st}$and $V_{ME}^{2nd}$respectively measured in the experimental set-up shown in Fig. 1(a).

To understand the complicated experimental results better, we analytically replicated the measurements based on the circuit model proposed in Ref [10] using MATLAB. Fig. S3(c) shows the theoretical magnetization in the y-direction (m_y_) which is assumed to be a sigmoidal function (tan-hyperbolic) of the magnetic field (H_y_) applied in the y-direction, closely matching the experimentally obtained M-H curve shown in Fig. 1(b). As in the experiments, the total magnetic field (H_y_) at time (t) is $H_{DC}+H_{_{AC}}*\sin(wt)$ where ω is the modulation frequency of the AC magnetic field with an amplitude H_AC_, and H_DC_ is the DC field. At a given time t, the magnetization is calculated as:

$m_{y}(t)=f(H_{DC}+H_{AC}*\sin(wt))$ (A1)

where f is the tan-hyperbolic function defined above. Since the total magnetization$m_{x}^{2}(t)+m_{y}^{2}(t)$ remains 1 for all applied magnetic fields in the X-Y plane, we define the pseudo-magnetization as a function of $t$ for different $H_{DC}$ ranging from -100 to +100 Oe as:

$\begin{matrix} \mu\equiv m_{x}^{2}(t)-m_{y}^{2}(t)=1-2*m_{y}^{2}(t) \\ =1-2*f(H_{DC}+H_{AC}*\sin(wt)) \end{matrix}$ (A2)

According to the analysis in Ref [10], the voltage generated due to the magnetoelectric effect is calculated by

$V_{ME}(t)=v_{m}*(m_{x}^{2}(t)-m_{y}^{2}(t))$ (A3)

where $v_{m}$ is the back-voltage constant determined by the coupling strength between the PE and FM layers. Figure S3(d) shows the results of V_ME_ as a function of time for H_AC_ = 4Oe with different H_DC_. The 1^st^ and 2^nd^ harmonic of V_ME_ extracted using Eq. (1) and Eq. (2) for different H_DC_ are plotted in Fig. S3(e).

As apparent, both the 1^st^ and 2^nd^ harmonics of the experimental and analytical results show a good qualitative match, which indicates that the simple analytical analysis can be used to explain the experimentally observed ME effect. Note that due to the hysteresis in the experimental M-H curve in Fig. 1(b) that might be a result of the hard axis not being perfectly aligned with the [01-1] crystalline direction, both harmonics curves are shifted along the x-axis depending on the direction of the H_DC_ sweep. It is worth to mention that in the analysis from above, V_ME_(t) is assumed to be too small to affect the magnetic properties of the CoFeB film. Without this assumption, one has to self-consistently solve problem, where the change in the magnetic properties is generating V_ME_ and the generated V_ME_ is affecting the magnetic properties. This approach has been used in the main text by utilizing the circuit shown in Fig. 2(b). The above assumption of V_ME_(t) being small is valid for the measurements in Fig. S3 since to change the M-H curves, at least tens of volts are required as shown in Fig. 3 and Fig. S2, while the voltage -V_ME_- generated by the AC magnetic field is only in the range of a few hundred microvolts.

IV: SIMULTANEOUS ME WRITE AND READ OPERATIONS


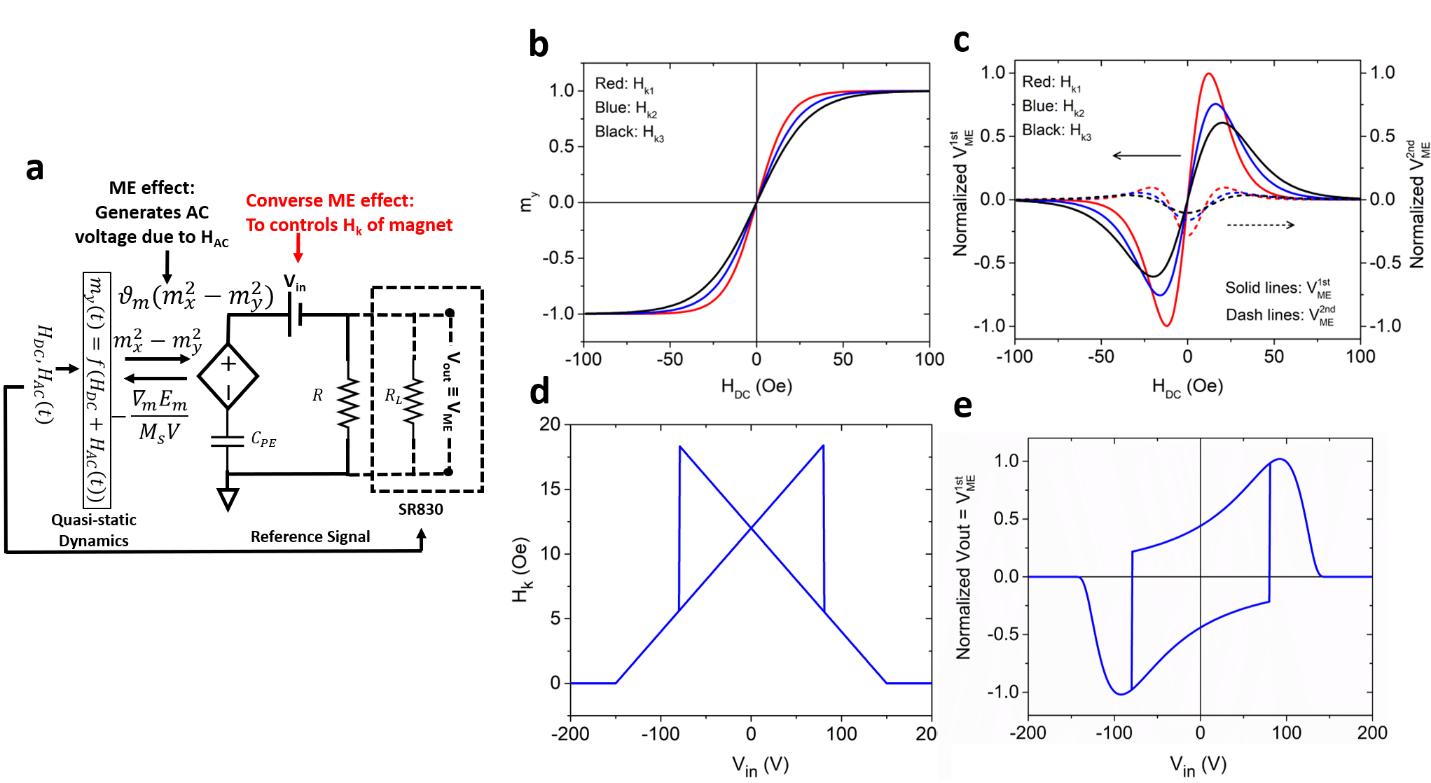


FIG. S4. (a) Circuit model of the simultaneous ME write and read operations. (b) Theoretical M-H loops of the [01-1] direction for different H_k_ with H_k1_ < H_k2_ < H_k3_; (c) The first and second harmonic of V_ME_, i.e. $V_{ME}^{1st}$ and $V_{ME}^{2nd}$corresponding to different H_k_ values (d) The relation between H_k_ of the magnetic film and the external DC voltage used in the simulation. (e) The relation between V_in_ and V_out_ ≡ $V_{ME}^{1st}$ when H_DC_ = 10Oe, H_AC_ = 10Oe and the frequency of the AC field is 112Hz.

Figure S4 shows simulation results on the simultaneous ME write and read operations using the same method as discussed above in Appendix C. The circuit model is shown in Fig. S4(a) – the magnetic state of the FM layer is controlled by the input voltage V_in_ and read out by the induced magnetoelectric voltage. To measure the magnetoelectric voltage using a lock-in amplifier SR830, a resistor R with a resistance value similar to the impedance of the piezoelectric capacitor is connected in series in the circuit. V_out_ ≡ $V_{ME}$ measured across R is used to read the magnetic information in the FM layer. Figure S4(b) is the theoretical M-H loops in the Y direction for different H_k_ (H_k1_ < H_k2_ < H_k3_) in line with the experimental results on ME write operation discussed in Fig. S2, however without hysteresis. The corresponding first and second harmonic of V_ME_ i.e. $V_{ME}^{1st}$ and $V_{ME}^{2nd}$are shown in Fig. S4(c). Figure S4(d) shows the relation between H_k_ of the magnetic film and the external DC voltages used in the simulation based on the analysis in Fig. S2, i.e. H_k_ in the Y axis decreases under large positive and negative voltages because the tensile strain in the Y direction and compressive strain in the X direction favors a magnetization along the Y direction. The butterfly-like hysteresis is in consistent with the butterfly-like trend of induced in-plane strain in the PMN-PT [9]. Accordingly, the relation between V_in_ and V_out_ ≡ $V_{ME}^{1st}$ is illustrated in Fig. S4(e) when H_DC_ = 10Oe, H_AC_ = 10Oe and the frequency of the AC field is 112Hz. This set of results qualitatively matches with both the experimental and SPICE simulation results shown in Fig. 4.


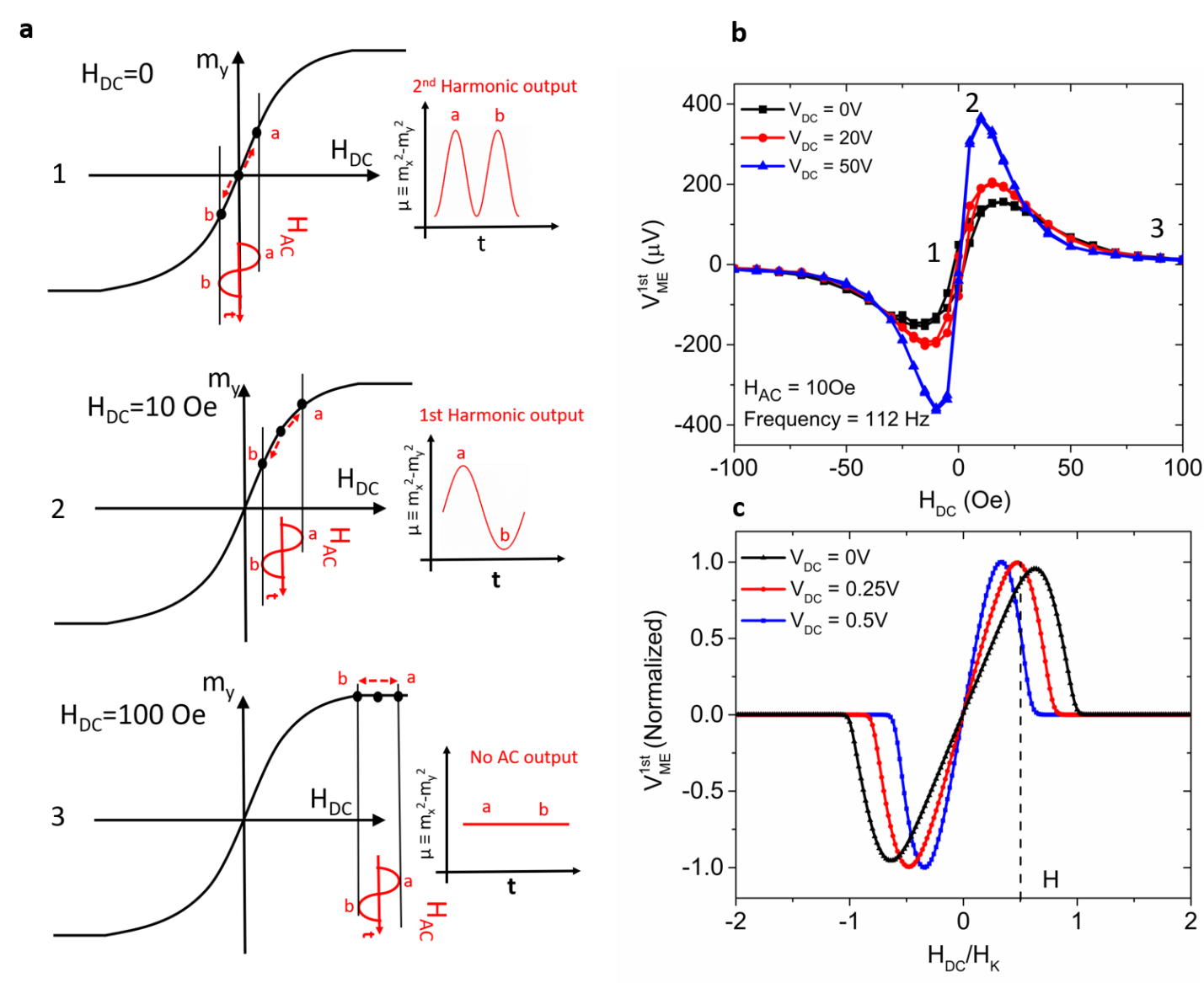


FIG. S5. (a) Schematic diagrams of the magnetization oscillation in the presence of an AC magnetic field applied on top of different DC magnetic fields in the [01-1] direction and the corresponding AC outputs. Case 1-3 illustrate the situations when H_DC_ = 0Oe, 10Oe and 100Oe respectively. (b) Experimental results on $V_{ME}^{1st}$ measured across R with an AC magnetic field superimposed on a DC magnetic field along the [01-1] direction when V_DC_ = 0V, 20V and 50V. States 1-3 correspond to the cases 1-3 in (a). (c) Simulation results of the normalized in-phase component of the AC voltage $V_{ME}^{1st}$ as a function of DC magnetic fields that is read out from the circuit in Fig. 2(b).

As discussed in the main text, V_ME_ is measured in the presence of an AC magnetic field applied on top of a DC magnetic field in the [01-1] direction using the experimental set-up shown in Fig. 2(a). Fig. S5(a) shows how the magnetization (m_y_) oscillates in the presence of an AC magnetic field applied on top of different DC magnetic fields in the [01-1] direction and the corresponding AC outputs. Based on the analysis in Fig. 1, the hard axis lies in the [01-1] direction when V_DC_ = 0V. Thus, the magnetization in the y-direction (m_y_) is approximately a sigmoidal function (tan-hyperbolic) of the magnetic field applied in the y-direction. In case 1 of Fig. S5(a), when H_DC_=0Oe, applying H_AC_, results in m_y_ and thus the pseudo-magnetization$\mu=m_{x}^{2}-m_{y}^{2}$to oscillate between points a and b. The oscillation frequency of$\mu=m_{x}^{2}-m_{y}^{2}$is twice that of H_AC_ with µ(a) = µ(b). According to Eq. (1) and (2), the 1^st^ harmonic of V_ME_ is thus zero and the AC output in case 1 only has contributions to the 2^nd^ harmonic of V_ME_ (see also Fig. S3(b) and (e)). However, for a finite H_DC_ (e.g. 10 Oe), the pseudo-magnetization oscillates with the same frequency as H_AC_. Thus,$\mu=m_{x}^{2}-m_{y}^{2}$is different for points a and b and a finite 1^st^ harmonic output signal is generated as shown in case 2 of Fig. S5(a). Last, for H_DC_=100Oe, m_y_ is in the saturation region and hence neither the magnetization nor the pseudo-magnetization oscillate with H_AC_, which results in a zero AC output signal. The experimental results in Fig. S5(b) follow this trend from case 1 to 2 and 3. Note that for a given H_AC_, the oscillation amplitude of m_y_ and µ is dependent of the slope of the m_y_-H_DC_, i.e. H_k_. Since V_DC_ applied across the PE-ME structure modifies H_k_ of the magnetic film, the AC output signal is expected to be different for different V_DC_-values. Positive V_DC_-values result in steeper m_y_-H_DC_ curves as shown in Fig. S4(b) and (c) and hence increase the oscillation amplitude of$\mu=m_{x}^{2}-m_{y}^{2}$for a given H_AC_, which results in a higher peak amplitude of V_ME_.

Figure S5(b) shows experimental results on the relationship between the first harmonic V_ME_ i.e. $V_{ME}^{1st}$and H_DC_ when V_DC_= 0V, 20V and 50V. The amplitude of the AC magnetic field is 10Oe and the frequency is 112Hz. The experimental results clearly show that both, the peak amplitude (V_P_) and the position of the peak (H_P_) are different for different V_DC_ following a trend: VP_50V > VP_20V > VP_0V and HP_50V < HP_20V < HP_0V, which corresponds to the different H_k_ of the CoFeB film caused by V_DC_.

Furthermore, the experimental results are described qualitatively by theoretical simulations using the equivalent circuit shown in Fig. 2(b). According to the M-H loops shown in Fig. 3(c), when V_DC_ increases from 0V to 0.5V, the magnetization in the [01-1] directions becomes easier which is consistent with the analysis in Fig. S2. From Eq. (1), the first harmonic in-phase load voltage$V_{ME}^{1st}$is calculated using our SPICE-model as a function of H_DC_ at different V_DC_ inputs. The corresponding results are shown in Fig. S5(c). The trends for a change in peak amplitude and peak position are qualitatively similar with the experiment results, i.e. higher V_P_ and smaller H_P_ for smaller H_k_.


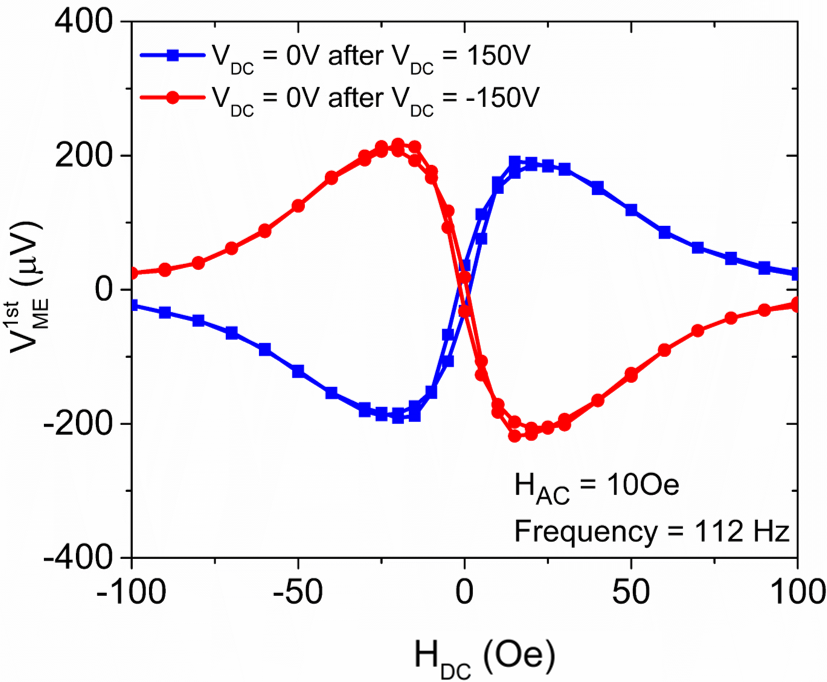


FIG. S6. The relation between $V_{ME}^{1st}$ and H_DC_ when V_DC_ = 0V after the device had been conditioned by applying a V_DC_ = 150V and -150V respectively.

To study the relationship between the piezoelectric polarization P and V_ME_, the PMN-PT was poled in both positive and negative [011] direction with large V_DC_. In Fig. S6, the blue and red lines are $V_{ME}^{1st}$ when V_DC_ = 0V after applying +150V and -150V respectively. The $V_{ME}^{1st}$ in these two cases has the same magnitude but opposite signs. In other words, V_ME_ (P) = -V_ME_(-P). This indicates that the hysteresis between the purple and orange lines in Fig. 4 is caused by the polarization hysteresis of the PMN-PT.

References

1. Liu, L.; Pai, C.F.; Li, Y.; Tseng, H.W.; Ralph, D.C. and Buhrman, R.A. Spin-torque switching with the giant spin Hall effect of tantalum. *Science* **2012**, *336*, 555-558.
2. Locatelli, N.; Cros, V. and Grollier, J. Spin-torque building blocks. *Nat. Mater.* **2014**, *13*, 11.
3. Liu, L.; Lee, O.J.; Gudmundsen, T.J.; Ralph, D.C. and Buhrman, R.A. Current-induced switching of perpendicularly magnetized magnetic layers using spin torque from the spin Hall effect. *Phys. Rev. Lett.* **2012**, *109*, 096602.
4. Wang, D.; Nordman, C.; Qian, Z.; Daughton, J.M. and Myers, J. Magnetostriction effect of amorphous CoFeB thin films and application in spin-dependent tunnel junctions. *J. Appl. Phys.* **2005**, *97*, 10C906.
5. Park, S.E. and Shrout, T.R. Ultrahigh strain and piezoelectric behavior in relaxor based ferroelectric single crystals. *J. Appl. Phys.* **1997**, *82*, 1804-1811.
6. Peng, J.; Luo, H.S.; Lin, D.; Xu, H.Q.; He, T.H. and Jin, W.Q. Orientation dependence of transverse piezoelectric properties of 0.70Pb(Mg_1/3_ Nb_2/3_)O_3_-0.30PbTiO_3_ single crystals. *Appl. Phys. Lett.* **2004**, *85*, 6221-6223.
7. Li, F.; Zhang, S.; Xu, Z.; Wei, X.; Luo, J. and Shrout, T.R. Composition and phase dependence of the intrinsic and extrinsic piezoelectric activity of domain engineered (1− x) Pb(Mg_1/3_Nb_2/3_)O_3_− x PbTiO_3_ crystals. *J. Appl. Phys*. **2010**, *108*, 034106.
8. Sun, E.; Zhang, S.; Luo, J.; Shrout, T.R. and Cao, W. Elastic, dielectric, and piezoelectric constants of Pb (In_1/2_Nb_1/2_)O_3_–Pb(Mg_1/3_Nb_2/3_)O_3_–PbTiO_3_ single crystal poled along [011] c. *Appl. Phys. Lett.* **2010**, *97*, 032902.
9. Thiele, C.; Dörr, K.; Bilani, O.; Rödel, J. and Schultz, L. Influence of strain on the magnetization and magnetoelectric effect in La_0.7_A_0.3_MnO_3_/PMN− PT (001)(A= Sr, Ca).  *Phys. Rev. B* **2007**, *75*, 054408.
10. Camsari, K.Y.; Faria, R.; Hassan, O.; Sutton, B.M. and Datta, S. Equivalent circuit for magnetoelectric read and write operations. *Phys. Rev. Appl.* **2018**, *9*, 044020.
